# Exploring the Phytochemical Compositions, Antioxidant Activity, and Nutritional Potentials of Edible and Medicinal Mushrooms

**DOI:** 10.1155/2024/6660423

**Published:** 2024-05-29

**Authors:** Mohammed Al Qutaibi, Suresh R. Kagne

**Affiliations:** ^1^Department of Medical Microbiology, Faculty of Science, Ibb University, Ibb, Yemen; ^2^Department of Microbiology, Badrinarayan Barwale College, Dr. Babasaheb Ambedkar Marathwada University, Aurangabad 431001, India

## Abstract

Mushrooms are a valuable source of food and medicine that have been used for centuries in various cultures. They contain a variety of phytochemicals, such as terpenoids and polysaccharides, that exhibit diverse biological activities, such as antioxidant, anti-inflammatory, anticancer, antimicrobial, immunomodulatory, and antidiabetic effects. However, mushroom's phytochemical composition and bioactivity vary depending on their species, cultivation conditions, processing methods, and extraction techniques. Therefore, using reliable analytical methods and standardized protocols is important for systematically evaluating the quality and quantity of mushroom phytochemicals and their therapeutic potential. This review provides a bibliometric analysis of the recent literature on biological activities, highlights trends in the field, and highlights the countries and journals with the highest contribution. It also discusses the nutritional value of the total content of phenolic and other phytochemicals in some species of mushrooms.

## 1. Introduction

**Figure 1:**
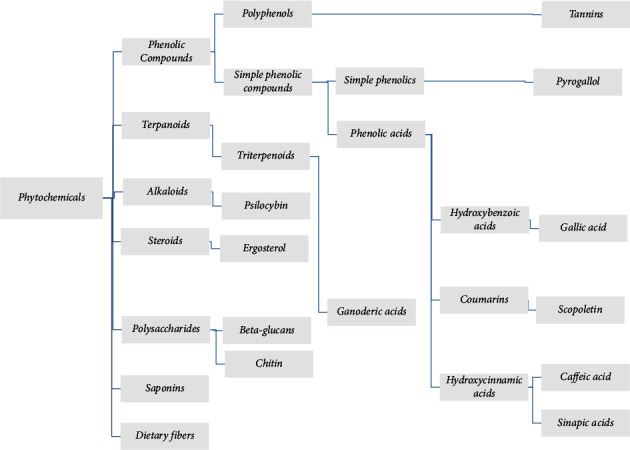
Some phytochemical compounds present in edible and medicinal mushrooms.

**Figure 2:**
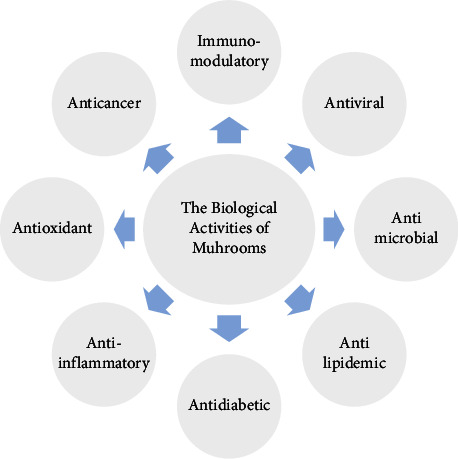
Some biological activities of mushrooms.

A mushroom is an epigeous, fleshy, fruiting body of macrofungus that typically grows in the soil or on a substrate such as organic matter [1]. Traditionally, mushrooms were used as a food source or remedy for their known healing properties. In ancient history, mushrooms were appreciated; for example, in ancient Egypt, only Pharaohs were the only people allowed to eat mushrooms, or “food of God,” as they call it [2]. Recently, there has been increasing interest in studying and exploring the biological activities of mushrooms [3]. The number of mushroom species was estimated to be around 1.5 million [4]. However, it is believed that the actual number is around 2.2 to 3.8 million worldwide [5]. Out of this number, only about 14000 species have been identified, 1400 of which are macrofungi [6], 1069 are considered edible [7], and 700 species were found to have therapeutic properties [8]. These therapeutic properties are attributed to the biologically active compounds in mushrooms [9], which could be used as a replacement for modern treatment methods, which have multiple side effects such as pain, high toxicity, emotional stress, and negative impact on physical and mental health [10]. The presence of secondary metabolites makes these macrofungi used in the pharmaceutical industry [11]. These secondary metabolites are known as “phytochemicals,” including terpenoids, polyphenols, steroids, alkaloids, polyketides, dietary fibres, and polysaccharides (especially beta-glucans) and are concentrated in the fruiting body of mushrooms [12].  Figure 1 shows some of the phytochemicals present in mushrooms. Phytochemicals possess a wide range of biological activities such as antioxidant, antimicrobial, antiviral, immunomodulatory, anticancer, hypocholesterolemic, anti-inflammatory, antiproliferative, anticoagulant, hepatoprotective, and hypoglycemic activities [13, 14].  Figure 2 shows some biological activities of edible and medicinal mushrooms.

The fruiting body is part of a mushroom, which grows on the soil and consists of a cap (*Pilus*), spore-forming part (*Sporophore*), and stipe (*Stem* or *Stalk*); the hypogeous part is known as mycelium (*hyphae*) [15]. These fruiting bodies are the edible parts of the mushrooms and contain all bioactive components such as phenolics [16], terpenoids [17], polysaccharides [18], lectins [19], antioxidants [20], and ergosterols [21, 22].

Triterpenoids [23], glucans [24], sesquiterpenoids [25], and glycoproteins [26] exhibit immune-modulatory and anticancer activity. Sterols, tocopherols, and indole compounds have antimicrobial and antioxidant properties [27].  Table 1 shows a list of important edible and medicinal mushrooms.

The study of mushrooms and their bioactive compounds holds immense significance for several reasons. One of these reasons is the biodiversity and unexplored potentials within the vast number of mushroom species [5] worldwide, only a few have been identified [6]. This vast, largely unexplored biodiversity represents a treasure trove of potential new compounds that could have significant therapeutic properties [28]. As we mentioned before, only 700 species of mushrooms are known to have therapeutic properties [8]. These mushrooms contain bioactive compounds that can potentially be used in the treatment of various diseases, offering a natural alternative to synthetic drugs [29, 30].

This review aims to highlight the role of the biologically active constituents (especially total phenolic content). The nutritional and medicinal properties include the nutritional content of proteins, carbohydrates, and vitamins in the most popular mushroom species. Furthermore, the review seeks to elucidate the advantages of certain edible and medicinal mushrooms, along with their relevant biological activities, including antioxidant and antimicrobial properties. In the current review, the methodology for the literature review was conducted to avoid misinformation and cover the literature gap. Additionally, the review's analysis of the phytochemical literature's bibliometrics helps to make a road map that directs readers to the best resources for learning more about this effort.

## 2. Literature Review Methodology

**Figure 3:**
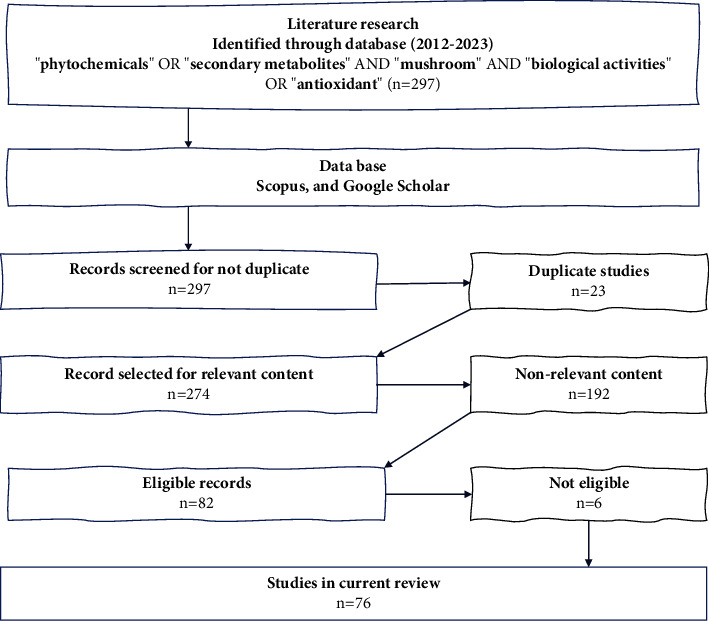
Literature review methodology.

Data were collected by searching scientific publications (reviews and/or research papers) in various databases, including Scopus and Google Scholar, using keywords such as “phytochemicals” OR “secondary metabolites” AND “mushroom” AND “biological activities” OR “antioxidant.” A total of 297 articles were the results of this search, and the record number was then screened for duplicate papers. Then, the records were screened for relevant (within the aims and scope of the review) or irrelevant contents, and eligible (research article, within the range 2012–2023, written in the English language) or ineligible contents.  Figure 3 illustrates this process [31, 32]. The total number of articles finally chosen and used to review the phytochemicals and the biological activity of edible and medicinal mushrooms was 76.

## 3. Bibliometric Analysis

Based on the Scopus database, the current study utilized bibliometric analysis to evaluate the global research trends on edible and medicinal mushrooms, their phytochemicals, and their medical application as antioxidants and antimicrobials. The publications (297) from the Scopus database were exported as a CSV file.

The bibliographic data analysis based on journals, countries, and keywords was done using the VOSviewer program, Leiden University, the Netherlands (version 1.6.19, for Microsoft Windows system) [33]. VOSviewer is a software tool developed for constructing and visualizing bibliometric networks. These networks can include elements such as journals, researchers, or individual publications, and they can be constructed based on citation, bibliographic coupling, cocitation, or co-authorship relations [33]. This program was used to import all the collected data from 283 documents, comprising 51 journals and 22 countries, for analysis. The software chose 51 journals (published 283 documents) for the productivity study.

**Figure 4:**
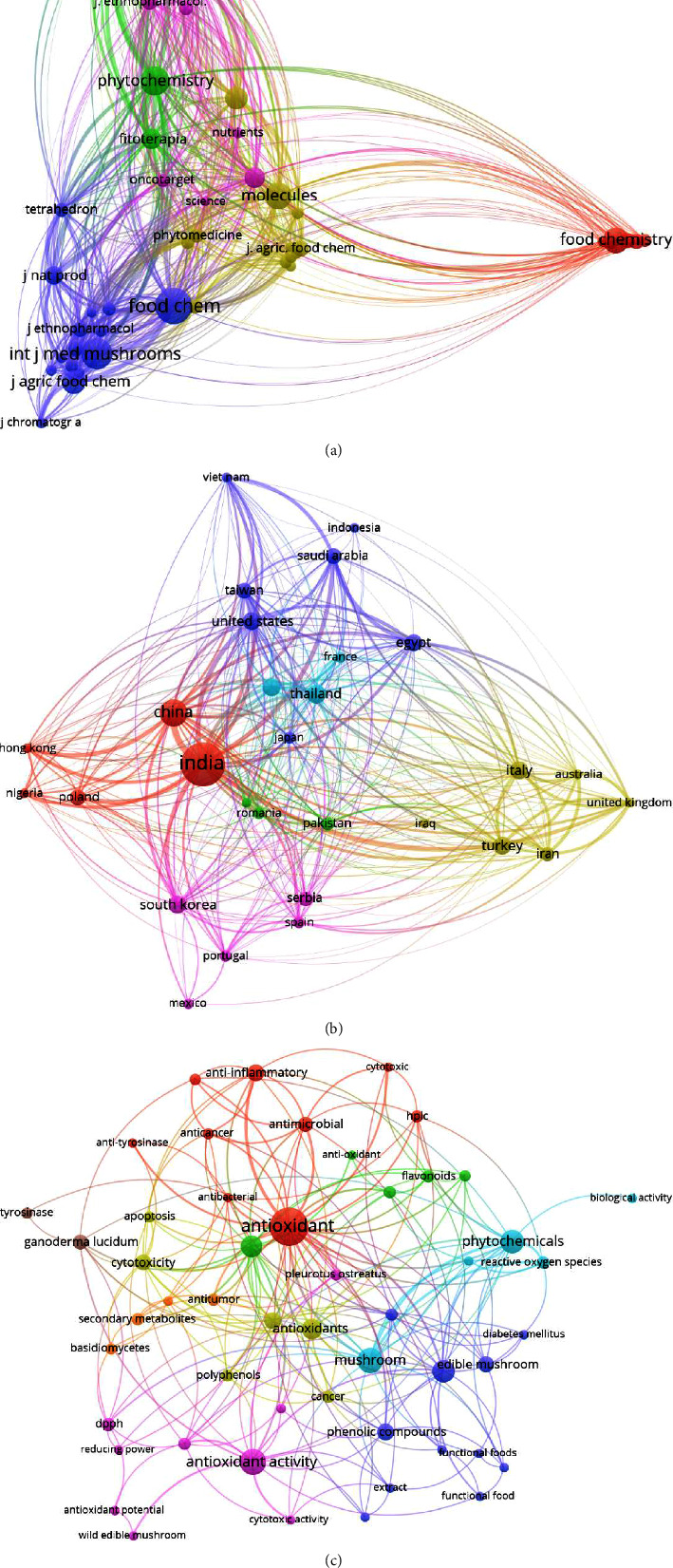
(a) Bibliometric analysis of the publications on edible and medicinal mushrooms based on the journals. (b) Bibliometric analysis of the publication on edible and medicinal mushrooms based on the countries. (c) Bibliometric analysis of the author keywords of edible and medicinal mushroom papers.

The most productive journals on mushroom phytochemicals and their biological activities were the following: *Phytochemistry, Food Chemistry, Journal of Natural Products, Fitoterapia, Journal of Agricultural and Food Chemistry, International Journal of Medicinal Mushrooms, Journal of Natural Products, Molecules, Chemical and Pharmaceutical Bulletin*, and *Plos One* (Figure 4(a)). The colour of the circles on the bibliographic map was utilized to help define the section for journals, where related keywords and journal contents were clustered together. Each circle's size represents the journal's strength in its total citations (TCs), total publications (TPs), and total link strength (TLS).

The countries that contributed the most were 22 in the subject of phytochemicals of edible and medicinal mushrooms and their biological activities. India, China, the United States, Saudi Arabia, Taiwan, Egypt, Malaysia, Italy, Turkey, and South Korea were the toppers (Figure 4(b)). This could be attributed to the biodiversity in these countries; most of them are considered the natural habitat of numerous mushroom species [6]. In many of these countries, particularly in Asia (such as China, India, South Korea, Malaysia, and Taiwan), mushrooms have been used for centuries in traditional medicine [34]. This traditional knowledge provides a valuable starting point for scientific research [35]. Other reasons could be that countries like the United States and Italy have advanced research infrastructures and invest heavily in research and development [35].

After analyzing the author's keywords' co-occurrence, the most mentioned mushrooms were *Pleurotus ostreatus, Agaricus bisporus, Ganoderma lucidum,* and *Inonotus obliquus.* The most mentioned activities in the author's keywords were antioxidant, anti-inflammatory, antimicrobial, anticancer, antitumor, and antidiabetic (Figure 4(c)).

## 4. Nutritional Properties

Edible and medicinal mushrooms are known for their nutritional properties and health benefits [36–38]. They are rich in proteins, carbohydrates, crude fibre, total phenolic compounds, fats, vitamins, and minerals [6, 39].

**Figure 5:**
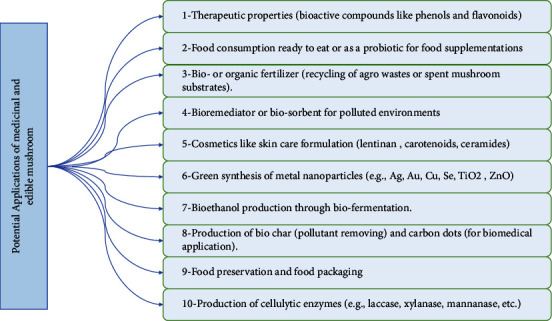
Potential applications of some edible and medicinal mushrooms and their by-products, which include using biologically active compounds in developing the nutraceutical and pharmaceutical formulations, in addition to applications such as bioremediation, animal feeds, energy production, fertilizers, bio-based materials, cosmetics, and cosmeceuticals.

The edible mushroom contains protein, amino acids, lipids, carbohydrates, mineral composition, and vitamins [15]. Because of their special flavour, fleshy texture, and nutrient-dense diet, mushrooms could be used as a replacement for meat. It is a good source of protein, dietary fibre, vitamins (B1, B2, C, niacin, folic acid, and ergosterol), and minerals (K, P, Na, Fe, and Ca), and it is low in fat [40]. The nutritional value of the mushroom species is determined by its chemical composition and concentration [15].  Figure 5 shows some applications of medicinal and edible mushrooms.

### 4.1. The Most Nutritious Mushroom Species

The *Pleurotus* species is one of the most nutritious mushroom species, containing low-fat, low-sodium, and cholesterol-free, water-soluble vitamins, proteins, minerals, chitin, and glucans [41]. *Pleurotus* sp. is considered to have 28.6 − 15.4% of proteins, 84.1 − 61.3% of carbohydrates, and 3–33.3% of dietary fibre [38]. Their protein content is higher than that of vegetables but less than that of mt and milk [38]. This species is a rich source of proteins and minerals (Na, Ca, P, Fe, and K) and vitamins (vitamin C and B complex) [38] The *Pleurotus* mushrooms harbour various active components including polysaccharides, dietary fibres, oligosaccharides, triterpenoids, peptides, proteins, alcohols and phenols, and mineral elements such as zinc, copper, iodine, selenium, and iron. Moreover, they also contain vitamins and amino acids [42, 43].

*Agaricus* mushrooms, including the common white mushroom (*A. bisporus*), are known for their nutritional properties. It contains calories, carbs, fibre, protein, vitamin D, selenium, phosphorus, and folate [44, 45]. In addition to these nutrients, *Agaricus* mushrooms contain compounds such as beta-glucans, ergosterol, and polyphenols that help reduce inflammation and improve cardiovascular health. They are also rich in amino acids and minerals, including calcium, phosphorus, zinc, potassium, and magnesium [46]. *A. blazei* Murill consisted of carbohydrates, ergosterol, fat, fibres, vitamins C, B1, B2, B9, B12, niacin, trace elements (Zn, Se), phenolics, and gallic acid [35].

Shiitake mushrooms (*Lentinula edodes*) are well known for their nutritional properties. The dried extracts of *L. edodes* comprise 58–60% carbohydrates, 20–23% proteins, 9–10% fibre, 4–5% ash, and 3–4% lipids [47]. The protein component of *L. edodes* differs from the protein in general food crops, including mainly albumin, gluten, and prolamin. Eight of the 18 amino acids in *L. edodes* are essential [48].

Lion's Mane mushrooms, or *Hericium erinaceus*, are among the most nutritious, with high proteins, carbohydrates, lipids, and dietary fibres [49]. Another medicinal species is *Ganoderma*, rich in proteins, fats, carbohydrates, and fibres, and minerals such as calcium, phosphorus, potassium, copper, iron, zinc, magnesium, and selenium [50].

### 4.2. Mushrooms Have High-Water Content

The high-water content of mushrooms makes them a low-energy diet, about 50–70 kcal/100 g [51]. Mushrooms are composed of approximately 90% water [51]. This high-water content contributes to their refreshing and hydrating quality [52]. Incorporating mushrooms into meals can help maintain overall hydration levels in the body [52]. The dietary considerations mean a low-calorie diet, hydration, and nutrient density [51]. Due to their low-calorie nature, mushrooms are an excellent choice for those aiming to manage their weight [51]. Proper hydration is essential for various bodily functions, including digestion, circulation, and temperature regulation [53]. Mushrooms offer essential nutrients such as vitamins (vitamins B and D), minerals (potassium, copper, iron), and antioxidants [40].

### 4.3. Nutritional Future Prospective of Mushrooms

Mushrooms can be used as a meat substitute in vegetarian and vegan dishes due to their texture and flavour [54]. They have a unique flavour known as umami, also found in meat, making them a satisfying substitute [55]. Oyster mushrooms can replace chicken [55], and they are more environmentally friendly than meat, as they require less land, water, and resources to grow [56]. Mushrooms are regarded as functional foods and nutraceuticals [57]. They support health and wellness and have potential as prophylactic or therapeutic agents [57]. Ongoing research explores the biotechnological and medicinal aspects of edible mushrooms cultivated worldwide [58]. These studies aim to uncover additional health benefits and applications [58].

## 5. Phytochemical Investigations

### 5.1. Total Phenolic Content

The gas chromatography-mass spectrometry (GCMS-MS) chromatograms were employed for the investigation of the phytochemical composition within the *Psilocybe natalensis* (magic mushroom) [77]. N-Hexadecoid was observed in all extractions (hot water, cold water, and ethanol) [77]. The ethanol extraction exhibited the greatest quantity of nanodecane and tetradecane, which possess antioxidation properties and anti-inflammation [77]. Advanced techniques with multiple exactions solvents give more reliable results [77]. A study assessed the TPC, glucose, and total protein composition of three different varieties of mushrooms, namely, *A. bisporus, P*. *columbinus,* and *L. sajor-caju* [78]. *A. bisporus* exhibited the highest TPC and total protein composition [78]. On the other hand, *P. columbinus* demonstrated the highest glucose content among the three species [78]. The TPC of five distinct mushroom species was analyzed, and it was discovered that *Astraeus hygrometricus*, a naturally occurring edible mushroom, exhibited the most elevated TPC among all the species that were investigated (*Serpula* sp.*, Phallus* sp.*, Tricholoma* sp., and *Lentinus* sp.) [79].

Five different species of mushrooms were subjected to examination in order to ascertain their phenolic profile utilizing the HPLC technique [80]. The findings demonstrated that *Hydnum repandum* exhibited the most noteworthy concentration of fumaric acid, rendering it the preeminent species among those evaluated [80]. Fumaric acid plays a crucial role as an intermediate in the citric acid cycle, essential for producing energy from carbohydrates, fats, and proteins in humans [81]. The phytochemicals found in H. erinaceus were analyzed using various extraction techniques (water, ethanol, acetonitrile, acetone, chloroform, and ethyl acetate) [82]. The findings revealed that this particular fungus possesses a considerable abundance of the TPC, protein, and nitrogen alongside a moderate quantity of tannins and saponins [82].

The total phenolic content of the Donko and Koshin varieties of *L. edodes* mushroom was assessed using two different extraction solvents [83]. The results indicated that, in the case of the Koshin var., the aqueous extract exhibited a greater concentration of the TPC than Donko's methanol and aqueous extracts [83]. The significance of these findings lies in the potential therapeutic applications of these mushrooms, especially in the context of increasing antibiotic resistance [83]. Similarly, the aqueous extract of *B. edulis* demonstrated the highest TPC [84]. It was significantly greater than the methanol extract of the same mushroom and the aqueous and methanol extracts of *Neoboletus luridiformis* [84]. The chemical composition of two *Pleurotus* mushrooms was evaluated by applying distinct solvents to extract them [85]. The methanolic, ethanolic, and aqueous extracts of *P. ostreatus* and *P. florida* possess alkaloids, tannins, and saponins [85]. The highest TPC concentration was observed in the methanolic extract in both *P. ostreatus* and *P. florida* compared to the other solvents, and the concentration was comparable [86]. On the other hand, steroids are exclusively identified in *P. ostreatus* within all extracts [85]. Meanwhile, anthraquinones and phlobatannins varied among the different extracts [85]. *P. ostreatus and P. florida* can be considered potential contenders for natural origins of antioxidants possessing considerable worth in the domains of pharmaceuticals and phytotherapy [85]. A research endeavour was undertaken to examine the characteristics of eleven types of mushrooms (*A. bisporus, Auricularia auricula-judae, L. sajor-caju, L. squarrosulus*, *Panus lecomtei, P. giganteus, P. ostreatus, P. pulmonarius, Polyporus arcularius, L. velutinus,* and *Lycoperdon scabrum*) gathered from the northeastern region of India [87]. The findings revealed that *L. sajor-caju* possessed the most elevated total phenolic content, trailed by *P. ostreatus* and *P. pulmonarius* [87].

#### 5.1.1. The Total Phenolic Content of Mushroom sp. under Special Conditions

An investigation was performed to ascertain the influence of various drying methods on the bioactive constituents within the *L*. *sajor-caju* mushroom [88]. The results indicated that the employment of solar drying within a temperature range of 45–47°C resulted in the greatest quantities of protein, beta-glucan, lipids, carbohydrates, and TPC (total phenolic content) [88]. This will help any future investigation on this species to consider using solar drying the mushroom sample [88]. The evaluation of the phenolic compounds in the tropical black bolete mushroom is considered an interesting preservation technique [89]. The results revealed that fresh samples exhibited the greatest quantity of the TPC, followed by frozen, dried, and brined samples, in that order [89]. These results highlight the importance of considering preservation techniques when assessing mushrooms' nutritional value and health benefits [89]. Using different supplements has an impact on the TPC [90]. A study assessed the overall phenolic content of *P. pulmonarius* cultivated with different supplements [90]. The findings revealed that the initial growth phase, when supplemented with gypsum, exhibited the greatest phenolic content [90]. The study suggests that the initial growth phase is the most important stage for phenolic production in *P. pulmonarius* [90]. Heat treatment can enhance the nutritional and medicinal value of Reishi mushrooms by increasing their phenolic content [91]. A study examined how heat treatment affected the phenolic content of dried fruiting bodies of Reishi medicinal mushrooms (*G*. *lingzhi*) [91]. The results showed that the samples subjected to heat treatment at 120°C and 150°C had the highest TPC, exceeding that of positive control (gallic acid) [91].  Table 2 shows a list of phytochemicals, different sample treatments, and medical applications of mushrooms.

#### 5.1.2. The Total Phenolic Content of Mushroom sp. Collected from Specific Regions

The phenolic content was assessed in four distinct mushroom species harvested from Turkey (*C*. *comatus, H. repandum, A*. *impudicus,* and *Sarcodon Imbricatus*); the findings revealed that the TPC was elevated in all species mentioned above [92]. Out of all the mushroom extracts examined, it was observed that *S*. *imbricatus* displayed the most substantial concentration of the TPC [92]. *S. imbricatus* is believed to possess the capacity to serve as a prospective reservoir for novel and alternative antioxidant substances within the realms of the food industry [92] *Armillaria mellea* and *M. procera* were procured from the countries of Morocco (MA) and Portugal (PT) [93]. After completing examinations, it was determined that the TPC was greater in *M. procera* obtained from Morocco [93]. Conversely, ascorbic acid, *β*-carotene, and lycopene exhibited higher levels in *A. mellea* sourced from Portugal [93]. The empirical data obtained in this comparative analysis unequivocally illustrate that the geographical, microclimatic, and edaphic circumstances of the collection site can influence the chemical composition, bioactive compounds, and antioxidant properties of the indigenous fungi [93]. Three indigenous edible fungi from the northeastern region of India underwent a comprehensive examination to determine their nutritional composition and ascertain the presence of phenolic compounds [94]. As a result of this analysis, it was discovered that *Ramaria thindii* possessed the most substantial quantity of total polyphenols, surpassing both *Macrocybe gigantea* and *Lactifluus leptomerus* in this regard [94]. The experimental findings demonstrate that each of the three wild consumable fungi is an outstanding reservoir of proteins, carbohydrates, vitamin C, and other vital minerals [94].

#### 5.1.3. Comparison Studies

In a comparison of two extraction methods, ethanol and aqueous extraction, the total phenolic content (TPC) of the inky cap mushroom (*C*. *radiata*) was analyzed [86]. The results indicated that the ethanol extract had a higher TPC than the aqueous extract [86]. The observed outcomes demonstrated that the polarity of the solvent had an impact on the TPC of the extracts derived from the *C. radiata* mushroom [86]. A comparative analysis was conducted to determine the highest levels of the TPC in commercially and laboratory-prepared extracts of six distinct species of mushrooms [95]. The findings revealed that Chaga, Maitake, Reishi, Shiitake, and Turkey Tail exhibited the highest TPC concentrations [95]. Another comparative analysis examined the polyphenolic composition of extracts from *B*. *edulis* and *Cantharellus cibarius* mushrooms collected in Romania [96]. The findings demonstrated that the highest TPC for *B. edulis* was observed in the shade-dried powder extract employing a methanol/water mixture as the extraction solvent [96]. Therefore, acidic water (CH_3_COOH _10%_) is the recommended solvent for extracting these bioactive compounds from *B. edulis* and *C. cibarius* mushrooms [96]. Conversely, in the case of *C. cibarius*, the fresh samples using pure methanol exhibited the highest TPC [96]. The beta-glucan content of five *Pleurotus* mushrooms was investigated, and the results indicated that *P. ostreatus* 5175 Florida had the highest beta-glucan content compared to the other samples tested [99]. In the in vivo assays, the extract from *P. ostreatus* Florida 5175 displayed the highest activity level in the NF-*κ*B inhibiting assay. This observed outcome can be attributed to the extract's notable content of total glucans [99]. The characteristics of *L*. *swartzii*, widely recognized as the Philippine sawgill mushroom, were assessed, encompassing an analysis of its phytochemical composition [100]. The findings unveiled that the fruiting bodies of this mushroom housed a considerable quantity of phenolic compounds, albeit in a lesser proportion than the quantity detected in the mycelia [100]. The current study has demonstrated that the mycelia and fruiting bodies of *L. swartzii* have the potential to be a natural source of phytochemicals that contribute to antioxidant and antidiabetic properties [100]. This finding strongly suggests that the chemical characteristics and biological activities are influenced not solely by the species, strain, culture media, and extraction solvent but also by the type of mushroom biomass used for extraction [100]. An investigation focused on analyzing the phytochemicals of the aqueous extracts derived from *L*. *edodes* and *P. ostreatus* [97]. It was discovered that *L. edodes* exhibited higher concentrations of the TPC than *P. ostreatus* [97]. Nevertheless, *L. edodes* also demonstrated greater total carbohydrates and proteins [97]. The aqueous extracts derived from mushrooms exhibit encouraging antiviral properties in the face of adenovirus type 7 and herpes simplex virus type-II [97]. The current study promotes the utilization of these fungi for pharmacological intentions in consideration of their minimal cytotoxicity [97]. The surrounding environments can affect the physiochemical and phytochemical properties of the same species of the mushroom [98]. The phytochemical characteristics of *G. lucidum* grown in the wild and those cultivated were evaluated [98]. The study revealed that the cultivated *G. lucidum* exhibited elevated concentrations of the TPC and water-soluble polysaccharides [98]. Conversely, the wild-grown *G. lucidum* displayed the most abundant terpenoid content [98].  Table 3 shows a list of phytochemicals, different sample treatments, and medical applications of mushrooms.

### 5.2. Other Phytochemicals

The secondary metabolites of four wild edible mushrooms grown in a tropical region were examined [101]. Qualitative tests indicated the presence of tannins in all of the species, while alkaloids and carotenoids were absent from all [101]. Triterpenoids were found in *A. auricula* and *Trametes versicolor*, while steroids were present in *Schizophyllum commune* and *Microporus xanthopus* but absent in the former two [101]. Saponins were detected in all mushrooms except *S. commune* [101]. Qualitative analysis was conducted to determine the presence of phytoactive compounds in the Reishi mushroom (*G. lucidum*). The ethanolic extract of this mushroom gives positive results for glycosides, terpenoids, phenols, and carbohydrates, while saponins and alkaloids were absent [102]. The HPLC-DAD-MS technique was used to analyze the extract of *P. columbinus* Quél. This analysis revealed the presence of gallic acid, catechin, hydroxytyrosol, chlorogenic acid, epicatechin, and benzoic acid in the mushroom extract [103]. These compounds are believed to contribute significantly to the antioxidant activity of this mushroom [103]. The qualitative presence of phytochemicals in *P. eryngii* mushrooms was investigated using various extraction methods [104]. Crude and butanol extracts yielded positive results for alkaloids, carbohydrates, saponins, lipids, sterols, glycosides, and terpenoids [104]. Nonetheless, some variations were observed in the results of other extracts [104]. Notably, tannins were absent in all the tested extracts [104]. The phytochemical composition of the chloroform extract from *Geastrum saccatum* was determined, and qualitative analysis revealed the presence of multiple active compounds, including phytosterols, saponins, phenolic compounds, tannins, proteins, glycosides, and terpenoids in comparison with the extracts obtained from methanol, ethyl acetate, acetone, petroleum ether, and hexane [105]. The polyphenol content of *T. versicolor* mushrooms was examined in methanol and water extracts, with the aqueous extract showing higher levels of total polyphenols (16.11 ± 0.14 mg GAE/g DW) compared to the methanol extract [106]. The findings also revealed the existence of heteropolysaccharides and small quantities of mannose, xylose, galactose, fucose, and glucuronic acid [106]. The exopolysaccharide from the *Calocybe* sp. mushroom was extracted and characterized, with ethanol extraction yielding higher than methanol and hot water extraction [107]. The carbohydrate content in the sample was also estimated [107]. The presence of active compounds in *L. edodes* mushrooms was investigated through hot water extraction and further assessment, revealing the presence of three types of sugar (neutral, uronic, and sulfated sugar) and a fair amount of protein and phenolic compounds [108]. The crude polysaccharide present in this mushroom serves as a reservoir of natural substances that possess beneficial properties for human health with the potential to be utilized in both the food and pharmaceutical sectors [108]. The phytochemical content of the *V. volvacea* mushroom extract was examined using different extraction methods, including hot water shaking (HS), microwave assistance (MA), and ultrasonic assistance (UA), with UA extraction yielding the highest total polysaccharide content and hot water shaking extract showing the highest beta-glucan content [109] The mycelial extracts of *P. djamor* and *P. florida* were examined to determine the presence of active components, and the phytochemical assessment of dichloromethane mycelial extracts revealed the presence of terpenoid compounds [110] and also a substantial quantity of the bioactive substances (such as anthraquinones, and terpenoids), all of which likely contributed to the observed biological efficacy [110]. The phytochemical properties of five species of wild mushrooms naturally grown in the Garhwal Himalayan region, India, were analyzed, with terpenoids and steroids found in all mushroom extracts and saponins not detected in any of them [111]. The presence of other compounds varied among different species [111]. The analysis was conducted to determine the total phenolic content of *B. edulis* and *N. luridiformis* [84]. It was observed that the aqueous extract of *B. edulis* displayed the highest TPC, surpassing both the methanol extract of the same mushroom and the aqueous and methanol extracts of *N. luridiformis* [84]. This result was deemed statistically significant [84]. The presence of phytochemicals in two species of *Ganoderma* mushroom was investigated [112]. Upon analyzing the methanolic extract, it was observed that the presence of carbohydrates, terpenes, and a small quantity of saponins was evident [112]. However, no alkaloids or flavonoids were detected [112]. Qualitative and quantitative methods were employed to assess the phytochemical content of the *H. erinaceus* mushroom, revealing a substantial quantity of phenolics and high levels of protein and nitrogen [82]. These compounds showed good antimicrobial activity against some human pathogens [82]. The extract of the *A. hygrometricus* mushroom was tested for phytochemicals, and it was found to contain phenolic compounds that directly contribute to its antioxidant, anticancer activity, and inhibition of atherosclerosis [113]. The *P. ostreatus* cv. Florida extract was found to have an abundance of phytochemicals, particularly amino acids, carbohydrates, and glycosides [114].

## 6. Antioxidant Activity

The radical scavenging activity of the magic mushroom *P. natalensis* was evaluated with the ABTS assay using different extracts [77]. Despite exhibiting the highest antioxidant efficacy compared to the other extracts, the ethanol extract exhibited lower values than the positive control, consisting of trolox and ascorbic acid [77]. The findings indicate that the concentrations employed for the water and ethanol extracts of *P. natalensis* are deemed safe and possess antioxidant and anti-inflammatory properties [77]. The antioxidant properties of three edible mushrooms belonging to the Agaricomycetes, specifically *P. columbinus, L. sajor-caju,* and *A. bisporus*, were subjected to investigation [78]. The outcomes derived from the DPPH assay disclosed that *P. columbinus* exhibited the highest activity [78]. Furthermore, the outcomes of the ABTS assay indicated that all three extracts surpassed Trolox, whereas the results obtained from the ORAC assay demonstrated that the *A. bisporus* extract displayed the lowest activity level [78]. The extracts derived from the three mushrooms possess considerable therapeutic potential for preventing and managing various illnesses [78]. The antioxidant potential of the wild edible and novel mushroom *A. hygrometricus* was evaluated [79]. The results obtained from the DPPH and ABTS assays indicated that the antioxidant efficacy of this mushroom was dosage-dependent [79]. Moreover, the antioxidant potentials present in the methanolic extract demonstrated a greater activity in the DPPH assay than in the ABTS assay [79]. It can be deduced that the methanolic extract of *A. hygrometricus* comprises a novel biomolecule that possesses appreciable antioxidant properties, which can potentially become a promising potent anticancer agent in the future [79]. The antioxidant activity of six mushrooms was examined, with Chaga, Maitake, Shiitake, Reishi, Lion's Mane, and Turkey Tail mushrooms being subjected to both commercially and laboratory-prepared extracts [95]. Five antioxidant assays were conducted, namely, ORAC, NanoCerac, DPPH, TPC, and FRAP. The results indicated a greater antioxidant activity for the laboratory-prepared extracts, particularly for Chaga and Maitake [95]. This study provides a foundation for future investigations into the antioxidant components of mushroom extracts generated using this technique and the variables influencing their durability and effectiveness [95]. The antioxidant efficiency of various mushroom extracts made in various ways was also compared [96]. *B. edulis* and *C. cibarius* were pulverized and extracted using acidic water, ethanol/water/-acetic acid, hexane, and diethyl ether [96]. The DPPH assay showed that the aqueous extract possessed a significant antioxidant capacity compared to other extracts [96]. The current investigation discloses that *B. edulis* and *C. cibarius* are the sources of polyphenolic compounds and display antioxidant properties, particularly when employing acidic water as a solvent [96].

The ABTS assay examined the antioxidant capacity of the isolated peptides from the split gill mushroom (*S. commune*) [115]. However, numerous studies have asserted that peptides with low molecular weight possess significant radical scavenging activity [116]. The results were rather unsatisfactory compared to the positive control, ascorbic acid [116]. The scavenging activity of the methanolic extract of *P. florida* was higher than other extracts when using the DPPH assay [117]. The bioactivity of the methanolic extract can be attributed to the presence of 3-methoxy-4-hydroxy cinnamic acid (PF5), which was obtained and purified from this particular mushroom [117]. The reducing power of the *P. florida* extracts was comparable to that of standard ascorbic acid [117]. PF5 holds great promise as a viable source of prospective antioxidant and anticancer compounds [117]. Moreover, it could potentially be effective in the role of preventive agents in combatting the pathogenesis of certain diseases [117]. Furthermore, it has been demonstrated that polysaccharides derived from deer tripe mushrooms (*A. delicata*) possess radical scavenging action [118]. The results from the DPPH and ABTS assays were relatively good but not comparable to vitamin C [118]. The study found that the crude polysaccharide has strong antioxidant properties, with a clear dose-effect relationship at lower concentrations [118]. The antioxidant activity of *C. indica* was assessed using various techniques such as radical scavenging and reduction ability [119]. The efficacy of radical scavenging was evaluated through the ABTS, DMPD, and superoxide radical scavenging tests [119]. The antioxidant activity of this mushroom extract against ABTS was found to be concentration-dependent [119]. However, the results were consistent with previous studies [119]. The aforementioned discoveries indicated that crude polysaccharides derived from *C. indica* have the potential to be deemed as a natural agent that inhibits oxidative damage and aids in the prevention of cancer [119]. Moreover, the bioactivity of *T. versicolor* exopolysaccharides was investigated, and the antioxidant activity was measured using the DPPH, ABTS, FRAP, and CUPRAC assays [106]. The outcomes revealed that the water extract exhibited higher antioxidant activity than the methanol extract [106]. Additionally, combining the macrofungal biomass and crude exocellular polysaccharide of *T. versicolor* presents natural dietary supplements with noteworthy health benefits [106]. The antioxidant activity of EPS isolated from the fruiting body of *Calocybe* species was evaluated using three different methods: FRAP, ABTS, and DPPH [107]. All three experiments demonstrated that *Cyclobe* species possess a high antioxidant activity [107]. The ABTS and DPPH values were higher than those reported in previous studies on other *Calocybe* species [107]. The antioxidant activity assays demonstrated the inhibitory effects of the *I. hispidus* extract against the ABTS and DPPH tests [120]. The extract performed better than commonly tested antioxidant chemicals [120]. According to this study, *I. hispidus* could be a promising source of bioactive compounds for health promotion and the development of functional foods [120]. The antioxidant activity of the *P. ostreatus* mushroom was assessed using both in vitro and in silico approaches [116]. The DPPH assays were used to evaluate the free radical scavenging activity [116]. As the concentration of the mushroom extract increased, its antioxidant activity also increased, although it remained relatively lower than that of the ascorbic acid [116]. *P. ostreatus* is rich in bioactive compounds, offering antioxidant, antibacterial, and anticancer properties [116]. Another dose-dependent outcome was the extraction of the *Laetiporus sulphureus* mushroom, which resulted in the extraction of the lovastatin compound [121]. The antioxidant activity of this compound showed a gradual increase with increasing concentration and was comparable to that of ascorbic acid [121].

In vitro assessment of the antioxidant activity of various *H. erinaceus* mushrooms was conducted using the DPPH free radical scavenging assay [82]. The aqueous extract exhibited the highest antioxidant activity, followed by the chloroform and ethyl acetate extracts [82]. *H. erinaceus* has the potential to serve as a crucial component of daily human dietary intake to protect from oxidative stress or viral invasion [82]. The polysaccharides derived from the *L. edodes* mushroom were evaluated for their antiradical activity using the DPPH and ABTS tests [108]. The radical scavenging ability showed a substantial effect at higher doses, although it was lower than ascorbic acid [108]. Furthermore, the EC_50_ value of this mushroom extract was lower than that of previously studied mushroom extracts [108]. *L. edodes* polysaccharides act as natural antioxidants, making them valuable for functional food and pharmaceutical applications [108]. The antioxidant capacities of three edible mushrooms, namely, *M. gigantea*, *L. leptomerus*, and *R. thindii*, were examined using the DPPH assay [94]. *M. gigantea* and *R. thindii* showed better results than *L. leptomerus* [94]. However, the antioxidant activity percentage of the three species was lower than that of ascorbic acid [94]. *R. thindii* demonstrated the most elevated cumulative phenolic content, following its utmost radical scavenging characteristics [94]. The ability of the wild mushroom *G. lucidum* to scavenge free radicals has been documented [10]. The high antioxidant activity of this mushroom can be attributed to its higher concentrations of triterpenoids and polyphenols [10]. Although the values did not align with those of the positive control, they were comparable to findings from other studies [10]. A study focused on investigating the antioxidant activity of the *A. hygrometricus* mushroom [113]. The excellent result can be attributed to its high phenolic content [113]. The mushroom also contains astragurkurol, which is rich in phenols [113]. Overall, this research sheds light on the potential health benefits of this mushroom and its natural antioxidant activity [113]. The antioxidant potential of five species of the *Pleurotus* mushroom was examined with the DPPH assay to assess their radical scavenging activity [99]. Among the five species (*P. flabellatus, P. pulmonarius, P. opuntiae, P. ostreatus* Sylvan Ivory, and *P. ostreatus* 5175 Florida), only *P. flabellatus* exhibited the highest antioxidant activity [99]. This result could be attributed to the highest levels of ergosterol and mannitol in *P. flabellatus* [99]. The biological activity of the *V. volvacea* mushroom was examined using three different extraction techniques: hot water shaking (HS), microwave assistance (MA), and ultrasonic assistance (UA) [109]. The strongest antioxidant activity was found in the HS extract [109] *V. volvacea* polysaccharides extracted with hot water shaking (HS) yield the highest amount of beta-glucan, which explains the extract's high antioxidant power [109]. Another advanced extraction technique was used when subcritical water extraction combined with a deep eutectic solvent to extract the polysaccharides from *L. edodes*, and their antioxidant activity was investigated [122]. This extraction technique demonstrates that the extract had a high level of antioxidant activity because this method helps to extract polysaccharides from this mushroom with higher scavenging capacity for DPPH, hydroxyl radicals, and hydrogen peroxide [122].

### 6.1. The Antioxidant Activity under Special Conditions

The antioxidant potential of the mushroom *L. sajor-caju* was investigated through various *drying methods* [88]. In order to determine the reducing power and radical scavenging efficacy, the FRAP and DPPH assays were employed, respectively [88]. Based on the findings, it was determined that the drying method does not impact the antioxidant activity [88]. Another investigation was done for the antioxidant activity of the polysaccharides obtained from antler mushrooms using three drying methods: hot air drying, vacuum drying, and freeze-drying [123]. This investigation yielded three polysaccharides (AMP-H, AMP-V, and AMP-F) that exhibited antioxidant activity and radical scavenging potential with a concentration-dependent effect [123]. In contrast to the previous study, the scavenging capacities of the DPPH radical were impacted by the diverse drying methods due to the alterations in the physicochemical properties of the polysaccharide samples [123]. A comparison was made regarding the antioxidant capabilities of tropical black bolete mushrooms using various *preservation methods* [89]. The findings from the DPPH and ABTS assays revealed that fresh, frozen, and dried sample extracts demonstrated higher activity compared to brined sample extracts [89]. Conversely, the brined sample extract displayed greater reduction potency than the other extracts with the FRAP assay [89]. Freezing is an effective preservation method that minimally alters the nutritional composition, phenolic compounds, and antioxidant properties even after one year in storage [89]. A study investigated the antioxidant activity of *P. pulmonarius* grown on sawdust with different supplementations [90]. The utilization of the DPPH assay demonstrated that the mushroom's fruiting body recovered from each flush revealed that zeolite treatment exhibited the highest radical scavenging activity [90]. Implementing zeolite augmented the production of mushrooms and the effectiveness of their biological processes [90]. Additionally, it triggered an elevated degree of antioxidant value in terms of radical scavenging activity and phenolic content [90]. A study focused on examining the impact of *heat treatment* on the dried fruiting bodies of *G. lingzhi* and its potential as an antioxidant [91]. The results indicated that the antioxidant capacity of the heat-treated samples was higher than that of the untreated samples, as determined by the DPPH and ABTS assays [91]. The study confirms that heat treatment enhances Reishi mushroom's beta-glucan solubility, antioxidant capacity, and lactogenic properties [91].

### 6.2. The Antioxidant Activity of Mushroom sp. Collected from Specific Regions

In Turkey, five species of mushrooms (*Daedalea quercina, H. repandum, I. radiatus*, *Omphalotus olearius*, and *S. commune*) were investigated for their antioxidant efficacy [80]. In order to evaluate their antioxidant activity, five assays were employed, including *β*-carotene-linoleic acid, DPPH, ABTS, CUPRAC, and metal chelating activity assays [80]. The methanol extract of *I. radiatus* and *H. repandum* exhibited the highest antioxidant efficacy in *β*-carotene-linoleic, DPPH, ABTS, and CUPRAC assays compared to the others [80]. The metal chelating assay revealed that the hexane extract of *S. commune* exhibited the highest antioxidant activity [80]. The antioxidant efficacy of two mushrooms, *A. mellea* and *M. procera* collected from Morocco (MA) and Portugal (PT), was studied. The results of the DPPH assay exhibited that *A. mellea* from PT has the strongest efficacy [93]. Statistically, the findings of the antioxidant outcomes revealed a marginal dissimilarity between mushrooms derived from MA and PT [93]. Wild mushrooms' chemical composition and antioxidant activity are varied based on geographic and climatic conditions [93]. Furthermore, an assessment was conducted to evaluate the antioxidant potential of dry fruiting bodies of wild edible mushrooms consumed in northeast India [87]. The findings indicated that *L. sajor-caju and L. squarrosulus* exhibited a high radical scavenging activity [87]. The nutritional value of wild edible fungi could help improve food security and nutrition for northeast India's indigenous populations, especially those following a vegetarian diet [87]. The antioxidant efficacy of wild mushrooms growing in tropical areas was also determined (*A. auricula, M. xanthopus*, *T. versicolor*, and *S. commune*) [101]. Compared to the positive control (vitamin C), only *S. commune* demonstrated a moderate radical scavenging activity [101]. Despite identifying secondary metabolites, the activities of *A. auricula, M. xanthopus,* and *T. versicolor* were weak [101]. Another study explored the antioxidant activities of the Philippine sawgill mushroom *L. swartzii* [100]. The findings from the DPPH assay revealed that the mycelial extract displayed a greater radical scavenging activity than fruiting bodies [100]. However, compared to the positive control (gallic acid and catechin), both the mycelial and fruiting body extracts demonstrated only moderate antioxidant potential [100].

## 7. Dosage-Dependent Aspect

Numerous studies mentioned that the antioxidant activity of some mushrooms can be dosage-dependent. Antioxidants, such as phytochemicals, can act bidirectionally by increasing or decreasing oxidative stress [124]. This means that the effect of these antioxidants on oxidative stress can vary depending on the dosage [124]. For instance, some studies have shown that certain antioxidants exhibit high reactive oxygen species (ROS) scavenging activity at low concentrations [124]. Implications of concentration-dependent antioxidant activity are significant in various fields, especially in health and medicine [125]. For example, it has been observed that the antioxidant and prooxidant activity of several flavonoid compounds in an iron-induced lipid peroxidation system with cultured hepatocytes can be concentration-dependent [125]. This suggests that the concentration of antioxidants can influence their effectiveness and role as either antioxidants or prooxidants [125]. Moreover, antioxidants have been demonstrated in vitro to have relevant pharmacological properties through their interaction with enzymes, transcription factors, and other proteins [126]. These interactions can be particularly relevant for preventing degenerative diseases of the central nervous system, cancer, and metabolic or immune diseases [126].

## 8. Conclusion

Mushrooms are gaining more interest globally for their health and nutritional values. They are highly nutritious and versatile food that also offers a variety of medicinal benefits. They are low in calories and fat but high in protein, fibre, vitamins, and minerals. Furthermore, they are rich in phytochemicals, including phenolics, beta-glucans, ergothioneine, and other compounds, which have been shown to have antioxidant, anti-inflammatory, and immune-boosting properties. The antioxidant activity of some mushroom species can be a powerful agent against inflammations and cancers. Mushrooms are indeed unique and beneficial in nutritional value and medicinal properties. These unique characteristics make mushrooms a subject of increasing interest in various fields, from nutrition and medicine to environmental science and material engineering. Mushrooms possess a fascinating array of properties that set them apart and contribute to their health benefits. They are nutritional powerhouses that provide immune support and are rich in antioxidants, which help combat oxidative stress and promote overall well-being. Mushrooms support a healthy inflammation response in the body, contain compounds that assist in balancing blood sugar levels, support brain function and neuron generation, and boost energy levels and stamina.

Additionally, these properties make mushrooms a promising food for preventing and managing various chronic diseases, such as cancer, diabetes, and cardiovascular disease. The potential of mushrooms is vast and still largely untapped, making them a fascinating area of study and application. However, further research is needed to fully understand the mechanisms behind these health benefits and to develop more targeted and effective mushroom-based therapies, such as exploring the molecular mechanisms of mushroom bioactivities, developing novel mushroom-based nanomaterials and drug delivery systems, and evaluating the clinical efficacy and safety of mushroom products. In general, incorporating mushrooms into a balanced and varied diet can provide significant nutritional and medicinal benefits.

## Figures and Tables

**Table 1:** List of some important edible and medicinal mushrooms.

S. no.	Scientific name	Common name	Photos		References
1	*A*. *bisporus*	Button mushroom	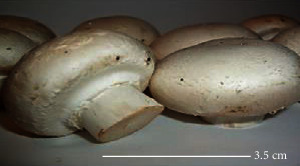		[59]

2	*P. ostreatus*	Oyster mushroom	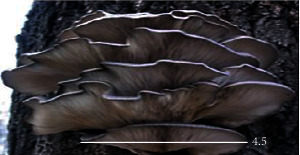		[60]

3	*G*. *lucidum*	Reishi mushroom	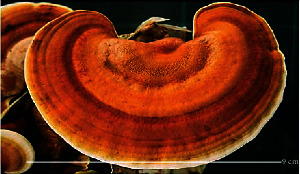		[61]

4	*A*. *blazei*	Royal sun Agaricus	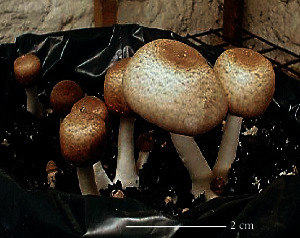		[61]

5	*Calocybe indica*	Milky white mushroom	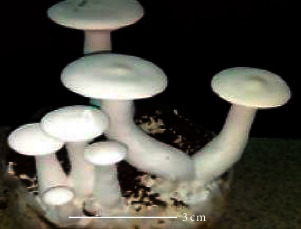		[62]

6	*L*. *edodes*	Shiitake mushroom	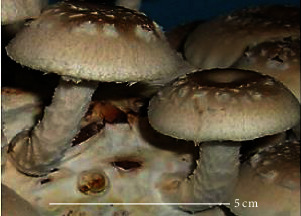	[63]	

7	*Flammulina velutipes*	Enoki mushroom	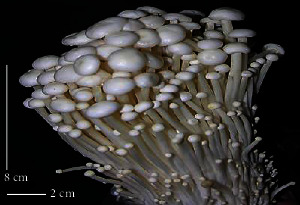		[64]

8	*Volvariella volvacea*	Straw mushroom	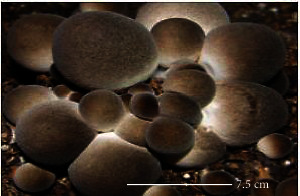		[65]

9	*H*. *erinaceus*	Lion's mane mushroom	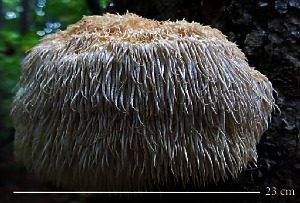		[66]

10	*Termitomyces heimii*	Termitomyces mushroom	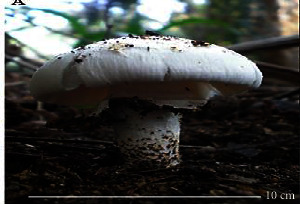		[67]

11	*L*. *sajor-caju*	Formerly known as *Pleurotus sajor-caju*	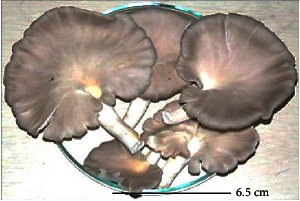		[68]

12	*P. florida*	White oyster	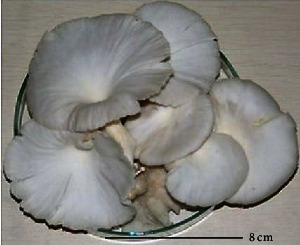		[68]

13	*P*. *djamor*	Pink oyster mushroom	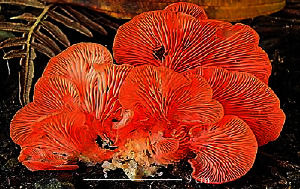		[69]

14	*P*. *eryngii*	King trumpet	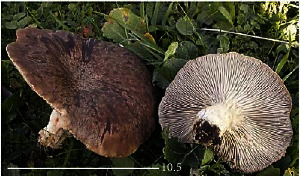		[70]

15	*Macrolepiota procera*	Florina	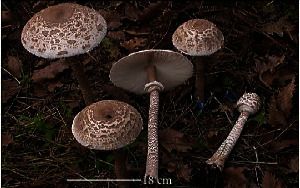		[70]

16	*Grifola frondosa*	Maitake mushroom	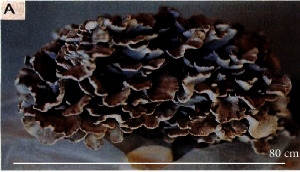		[71]

17	*Pholiota microspora*	Slippery mushroom	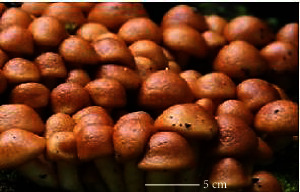		[72]

18	*Coprinus comatus*	Shaggy mane	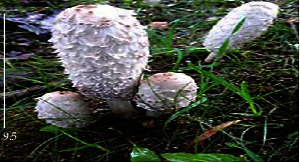		[73]

19	*Morachella esculenta*	Morels	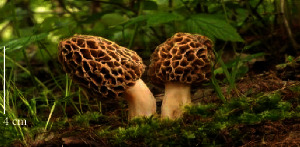		[74]

20	*Boletaceae boletales*	Boletes	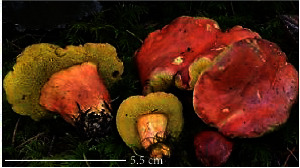		[75]

21	*Boletus edulis*	Porcini mushroom	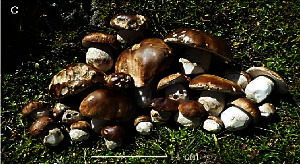		[76]

**Table 2:** Some phytochemicals, name of the mushroom, concentration, extraction solvent and/or drying method, and medical application.

S. no.		Name of the active compound	Mushroom	Concentration	Sample treatment	Medical application	References
1	Total phenolic content		*C. radiata*	32.27 ± 2.23 mg GAE^a^/g dry weight	Ethanol	Anticancer and antifungal	[86]
			*L. sajor-caju*	48.38 mg TAE^b^/gm	Solar drying	Bowel syndrome and colon cancer	[88]
			*A. bisporus*	27.45 ± 0.8 mg/g extract	Methanol	Antitumor, detoxicating	[78]
			*P. portentosus*	30.10 ± 1.04 mg GAE/g DW	Fresh sample	Antimicrobial, immunomodulatory	[89]
			*I. obliquus*	48.3 ± 3.9 *μ*mol GAE/mg	Aqueous extract	Antidiabetic, antioxidants	[95]
			*B. edulis, C. cibarius*	3.73 ± 0.007 0.79 ± 0.006 mg GAE/g DW	Aqueous extract	Antioxidant	[96]
			*G. lingzhi*	4.31 mg GAE/g DW	Heating 120–150°C	Anti-obesity, anti-inflammatory and prebiotic	[91]
			*L. edodes*	24.14 ± 1.01 mg/g extract	Aqueous extract	Hepatoprotective, antihyperglycemic	[97]
			*L. edodes* Koshin var	4.09 ± 0.59 mg GAE/g DW	Aqueous extract	Hepatoprotective, antihyperglycemic	[83]
			*S. imbricatus*	64.69 ± 3.25 mg GAE/g	Methanol	Scavenge free radicals	[92]
			*G. lucidum* godean	5.07 ± 0.39 mg GAE/g DW	Ethanol 70%	Antitumor, hepatoprotective, anti-inflammatory	[98]
			*B. edulis*	24.74 ± 1.11 mg GAE/g DW	Aqueous extract	Antioxidant, antimicrobial	[84]

2	Total protein content		*P. columbinus*	0.12 ± 0.004 mg/e extract	Aqueous extract	Antitumor, detoxicating	[78]
			*P. ostreatus*	0.1 ± 0.005 mg/e extract	Aqueous extract	Antihyperglycemic, immunomodulatory	[97]

3	Total carbohydrate content		*P. ostreatus*	76.56 ± 2.52 mg/g extract	Aqueous extract	Antimicrobial antiviral, hepatoprotective	[97]

4	Glucose content		*P. columbinus*	2.24 ± 3.98 mg/g extract	Aqueous extract	Antitumor, antiviral, antiparasitic, antibacterial	[87]

^a^Gallic acid equivalent, ^b^tannic acid equivalent.

**Table 3:** Some phytochemicals, name of the mushroom, qualitative results, extraction solvent and/or drying method, and medical application.

S. no.	Name of the active compound	Mushroom	Qualitative results	Sample treatment	Medical application	References
1	Carbohydrate	*L. sajor-caju*	+	Solar drying	Bowel syndrome and colon cancer	[88]
		*G. lucidum*	+	Ethanol	Antitumor	[102]
		*P. eryngii*	+	Crud and butanol extract	Antibacterial	[104]
		*Calocybe* sp.	+	Ethanol	Immunomodulatory	[107]
		*G. tuberculosum*	+	Methanol	Nephroprotective anticancer, antidiabetic, and antiviral	[112]
		*G. parvigibbosum*				
		*P. ostreatus* cv. Florida	+	Water: ethanol (1 : 1)	Antioxidant, antidiabetic, anthelmintic, antimicrobial and analgesic effects	[114]

2	Beta-glucan	*L. sajor-caju*	+	Solar drying	Bowel syndrome and colon cancer	[88]
		*V. volvacea*	+	Hot water shaking extract	Immunomodulation, anticancer, cardiovascular, antiviral, and antibacterial	[109]
		*P. ostreatus*	+	Methanol 80% chloroform	Anticancer, immunomodulatory antioxidant	[99]

3	Terpenoids	*P. djamor*	+	Dichloromethane mycelial extract	Anticancer, antioxidant, antitumor, anti-inflammatory, antimicrobial	[110]
		*P. florida*				
		*C. cibarius*	+	Ethanol	Anti-HIV, antitumor, antidiabetic, anticoagulant	[111]
		*A. auricula*	+	Ethanol	Antioxidant	[101]
		*T. versicolor*				
		*G. saccatum*	+	Chloroform extract	Anti-inflammatory antioxidant	[105]

4	Alkaloids	*P. ostreatus*	+	Methanolic, ethanolic, and aqueous	Antimicrobial, antioxidant	[85]
		*P. florida*				
		*P. eryngii*	+	Crud and butanol extract	Antibacterial	[104]

5	Tannins	*S. commune*	+	Ethanol	Antioxidant	[101]
		*M. xanthopus*				
		*A. auricula*				
		*T. versicolor*				
		*G. saccatum*	+	Chloroform extract	Anti-inflammatory antioxidant	[105]
		*H. erinaceus*	+	Methanol 80%	Antimicrobial, antioxidant	[82]
		*P. ostreatus*	+	Methanolic, ethanolic, and aqueous	Antimicrobial, antioxidant	[85]
		*P. florida*				

6	Saponins	*M. xanthopus*	+	Ethanol	Antioxidant	[101]
		*A. auricula*				
		*T. versicolor*				
		*P. ostreatus*	+	Methanolic, ethanolic, and aqueous	Antimicrobial, antioxidant	[85]
		*P. florida*				
		*G. tuberculosum*	+	Methanol	Nephroprotective anticancer, antidiabetic, and antiviral	[112]
		*G. parvigibbosum*				
		*P. eryngii*	+	Crud and butanol extract	Antibacterial	[104]
		*G. saccatum*	+	Chloroform extract	Anti-inflammatory antioxidant	[105]

7	n-hexadecoid	*P. natalensis*	+	Hot water, cold water, ethanol	Antioxidant anti-inflammatory	[77]
8	Nanodecane tetradecane			Ethanol		

9	Sterols	*P. eryngii*	+	Crud and butanol extract	Antibacterial	[104]
		*G. saccatum*	+	Chloroform extract	Anti-inflammatory antioxidant	[105]

10	Glycosides	*G. lucidum*	+	Ethanol	Antitumor	[102]
		*P. eryngii*	+	Crud and butanol extract	Antibacterial	[104]
		*G. saccatum*	+	Chloroform extract	Anti-inflammatory antioxidant	[105]
		*P. ostreatus cv.* Florida	+	Water: ethanol (1 : 1)	Antioxidant, antidiabetic, anthelmintic, antimicrobial, and analgesic effects	[114]

11	Lipids	*L. sajor-caju*	+	Solar drying	Bowel syndrome and colon cancer	[88]
		*P. eryngii*	+	Crud and butanol extract	Antibacterial	[104]

12	Anthraquinones and phlobatannins	*P. ostreatus*	+	Methanolic, ethanolic, and aqueous	Antimicrobial, antioxidant	[85]
		*P. florida*				

## Abbreviations

Vit. C: Vitamin C
AA: Ascorbic acid
TPC: Total phenolic content
EPS: Exopolysaccharide
EC50: Half maximal effective concentration
DPPH: 2,2-Diphenyl-1-picrylhydrazyl
PF5: 3-Methoxy-4-hydroxy cinnamic acid
ORAC: Oxygen radical absorbance capacity
CUPRAC: Cupric ion reducing antioxidant capacity
DMPD: N, N-Dimethyl-phenylenediamine.
